# Chiropractic students’ characteristics influencing confidence and competence in modulating spinal manipulation force–time characteristics of specific target forces: a secondary analysis of a cross-sectional study

**DOI:** 10.1186/s12998-025-00577-0

**Published:** 2025-04-27

**Authors:** Casper Nim, Nicole Smith, David Starmer, Simon Wang, Grand Choi, Akram Alayed, Jomana AlShareef, Angela Gnjatic, Keegan Sloan, Kitlyn Wong, Martha Funabashi

**Affiliations:** 1 Medical Research Unit, Spine Centre of Southern Denmark, University Hospital of Southern Denmark, Sygehusvej 24, 6000 Kolding, Denmark; 2 Department of Regional Health Research, University of Southern Denmark, Odense, Denmark; 3 Department of Sport Science and Clinical Biomechanics, University of Southern Denmark, Odense, Denmark; 4 Canadian Memorial Chiropractic College, Toronto, Canada; 5 Department of Chiropractic, Université du Québec à Trois-Rivières, Trois-Rivières, Canada; 6 Research Center, Parker University, Dallas, USA

**Keywords:** Spinal manipulation, Force-sensing table, Confidence, Teaching, Feedback

## Abstract

**Background:**

Although distinct, confidence and competence play a valuable role in healthcare education. For chiropractic students, both may be important in mastering motor skills required to perform spinal manipulative therapy (SMT). However, little is known about how individual factors influence students' confidence and competence. Better understanding of these associations would enable the development of tailored training. Therefore, this study aimed to investigate associations between demographics, anthropometrics, and prior SMT experience and confidence and competence in performing SMT with specific force–time characteristics in chiropractic students.

**Methods:**

This secondary analysis of a cross-sectional study involved 149 chiropractic students who performed SMT targeting specific peak thrust forces (200 N, 400 N, 800 N). Students were assessed for competence in force–time characteristics (preload, peak thrust force, time to peak force) using the force-sensing table technology, and self-reported their confidence in performing each characteristic. Demographics, anthropometrics, and SMT experience were collected and multivariable linear and logistic regressions were used to assess associations.

**Results:**

Confidence was higher in male students, students in later years of study, and those with more SMT experience. Competence in time to peak force was higher among males and third-year students, whereas males and taller students were more likely to reach the 800 N peak thrust force. No other associations were found for competencies.

**Conclusions:**

While certain demographic and experiential factors are associated with increased confidence, these do not consistently translate to competence in SMT force–time characteristics. Targeted training approaches that account for individual student factors to better support them in developing their SMT motor skills are needed.

## Background

Confidence and competence in delivering healthcare services is difficult to fully examine in a generalizable approach across different settings, in fact, it is even difficult to define exactly what is meant by these terms [1, 2]. In this study, we consider confidence to be defined by the belief in their ability to carry out a particular skill, and competence to be defined by their ability to perform that skill in practice [1, 2]. While it is not surprising that clinicians seek out opportunities to gain competence, it does appear that they also actively seek out opportunities to gain confidence [3]. Unfortunately, the relationship between confidence and competence is not straight forward, as higher confidence sometimes increases competence and other times it does not [3–8].

Therefore, the influence and development of confidence should at least be acknowledged in addition to competence for healthcare students during their education, when it comes to chiropractic we have limited knowledge about students’ confidence. Constructive feedback and guidance during theoretical and practical scenarios have been observed to be linked with both students’ increase in confidence and competence [9]. Another approach used simulation-based training, which demonstrated improved competence and confidence during simulated care activities [10].

In chiropractic education, one critical ability that is taught across educational institutions is mobilization of the spine most commonly using high-velocity, low-amplitude thrust or spinal manipulative therapy (SMT) [11, 12]. One simulation-based approach is the use of force-sensing devices to teach SMT techniques and motor skills. The force-sensing devices provide students with quantifiable measures of their SMT performance by giving visual feedback on the biomechanical aspects of their SMT, more specifically, force–time characteristics, such as preload forces, peak forces, and time to peak force [13–15]. Studies have shown that using these devices reduces students' variability when delivering SMT [13, 16–18]. While it may increase students’ competence in performing SMT, it also provides educators with a standardized and quantifiable approach of assessing such competence. Clearly, significant focus has been placed on optimizing SMT competence. However, there remains a notable lack of understanding regarding students’ confidence in applying SMT.

Arguably, confidence is a crucial element for students' success in clinical settings and may aid in fostering trust with patients [19, 20]. We recently published a study investigating the association between chiropractic students’ confidence and their competence in modulating SMT force–time characteristics [8]. We found that while students felt confident delivering lower forces (200 N and 400 N), their confidence significantly decreased at higher forces (800 N), mirroring their competence being challenged at this level. A positive association emerged between confidence and competence in performing specific SMT force–time characteristics, such as maintaining preload and a time-to-peak force below 150 ms. Interestingly, experience with force-sensing devices did not moderate this association, suggesting that confidence is independently associated with SMT competence.

To build on these new findings, it is crucial to understand the factors that may influence both confidence and competence in delivering SMT. There is still limited understanding of how individual differences, such as demographic factors (e.g., age, sex), anthropometric characteristics (e.g., height, weight), and prior SMT experience (e.g., year of study and time practicing with force-sensing devices) affect confidence and competence. Exploring these relationships could reveal whether specific intrinsic or experiential factors help develop confidence and competence in delivering SMT with specific force–time characteristics. This would provide a more comprehensive framework to guide training approaches within the biopsychosocial model. Specifically, a better understanding of these associations would enable educators to tailor training interventions, ensuring that all students—regardless of their background or physical characteristics—can develop the confidence and competence necessary for clinical success.

Therefore, the aim of this study was to investigate associations between demographic, anthropometric, and SMT experience factors and confidence and competence in delivering specific SMT force–time characteristics on mannikins.

## Methods

### Design

This is a secondary analysis of a cross-sectional study, registered prospectively at Open Science Framework (https://osf.io/6f7d5) [8]. This analysis follows the a-priori analysis plan, however, we had to make minor amendments to the protocol following the study, reported elsewhere [8]. For the current study, we increased the error rate of hitting the target to 75N instead of 50N, as this was more applicable to real life education and provided a more equal distribution of data. We adhered to the STrengthening the Reporting of OBservational studies in Epidemiology reporting guidelines [21]. This study was reviewed and approved by the Canadian Memorial Chiropractic College (CMCC) Research Ethics Board (REB approval # 2311B02). All participants reviewed and signed an electronic informed consent prior to participating in the study. The full study protocol can be seen elsewhere [8], below we provide an overview.

### Setting and study participants

Our study was conducted at CMCC’s Force-sensing Table Technology (FSTT®) laboratory in January 2024, in Toronto, Canada, enrolling first to third-year students without musculoskeletal issues. Recruitment was done with convenience sampling, using study posters, sign-up sheets, and word of mouth [8].

### Data collection

#### Study procedure

**Fig. 1:**
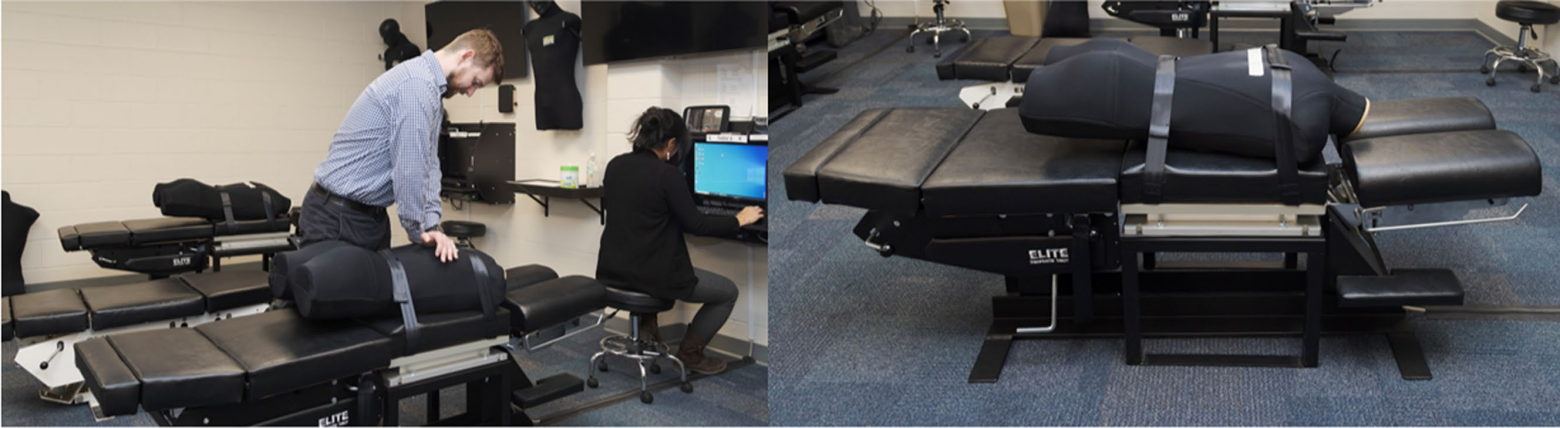
The study setup at the FSTT® laboratory [8]

Participants self-reported the demographic, anthropometric, and confidence in providing specific SMT force–time characteristics on an iPad (Apple, California, USA). All questionnaires were completed via the SurveyMonkey electronic data capture platform (Momentive Inc., California, USA). Following, participants were directed to a table, where a Human Analogue Mannikin (HAM®, CMCC, Toronto, ON, Canada) was positioned in a prone position on the Force-Sensing Table Technology (FSTT®, CMCC, Toronto, ON, Canada) and restrained by straps (Fig. 1).

Participants were asked to perform three thrusts with peak forces of 200N, 400N, and 800N within a ± 50 N range with a time to peak force < 150 ms in a random order using a concealed sequence that was determined a-priori [8]. To familiarize students with the procedures, three practice thrusts were allowed. Next, students were asked to define the preload they judged appropriate that should be consistent across all three trials. For each thrust force students provided two posterior-to-anterior, SMTs using their preferred hand contact (bilateral hypothenar, cross-bilateral hypothenar, or bilateral thenar). The students were able to select a table with a height that best fitted their preference [8] (Fig. 1).

## Variables

### Outcomes

#### SMT Force–time characteristics

All SMT force–time characteristics outcomes were measured through the FSTT® (see [8] for a detailed technical description). All outcomes were binary scored as *competent* or *not competent* in performing specific SMT force–time characteristics. This was derived based on whether they were able to modulate their SMT force–time characteristics within the following specified ranges:

- Pre-established preload force [N]—the force performed and held prior to the thrust force: Competent was considered if the preload before the three thrusts (200 N, 400 N, and 800 N) was consistent with the pre-established preload ± 75 N.
- Peak thrust forces [N]—the maximum force measured during the SMT: Competent was based on whether the peak thrust force was achieved ± 75 N (e.g., if participant targeted 400 N, competent was considered from 325 to 475 N).
- Time to peak force [ms]—the time between the take off and peak thrust force: Competent was considered if the time to peak force was below 150 ms.

The outcomes were automatically analyzed by the FSTT® software with standardized algorithms to identify the SMT force–time characteristics of interest. If failures occurred, data was reviewed manually and corrected in consensus with MF, NS, DS, and GC [8]. The FSTT® has demonstrated excellent reliability in measuring SMT force–time characteristics [22].

#### Confidence in providing specific SMT force–time characteristics

Participants were asked to indicate their level of confidence in being competent across the pre-established preload and the three SMT force–time characteristics targets (peak thrust force and time to peak force) using a 100mm electronic Visual Analogue Scale, where 0 corresponds to “not confident at all” and 100 corresponds to “completely confident” [15].

### Demographic and anthropometric factors

Participants provided self-reported information on the following characteristics, if they were in doubt a measuring tape and a scale were available:

- Sex [Male, Female]
- Age [Years]
- Height [Inches and reported in cm]
- Weight [Pounds and reported in kg]

### Experience factors

- Year of study [1st, 2nd, 3rd]
- Time spent in the FSTT® laboratory: Data on participants' laboratory attendance was obtained from the FSTT® current academic year attendance records (i.e., between June 2023 and January 2024). This data was reported in hours and calculated by summing all hours spent in the lab. This included open-lab, where students could log in and practice independently. Each login was estimated to correspond to one hour. Attendance in tutor-led laboratory sessions was measured as the proportion of hours attended, with 100% attendance equating to 52 h for first-year students, 70 h for second-year students, and 63 h for third-year students.

### Statistical analyses

This analysis included data on 149 participants from the original study; one participant had to be excluded as data indicated that the participant misunderstood the instructions [8]. We tabulated demographic, anthropometric, and experience factors using means and standard deviations or absolute and relative frequency as appropriate. We reported on SMT force–time characteristics and confidence outcomes using violin plots with embedded box plots stratified by competence to maintain preload, achieve target thrust forces, and meet the time to peak force requirement.

To assess associations we first scaled weight, height, and experience by divided with 10, meaning that the results were reported of 10 unit increases rather than 1-unit increases. We then conducted multiple linear regressions with confidence in maintaining preload, time to peak forces, and peak thrust force across 200N, 400N, and 800N as dependent variables. Independent variables included demographic, anthropometric, and experience factors, ensuring that weight and height were not adjusted for sex, years of study was not adjusted for age, and weight was not adjusted for height.

Next, we repeated this procedure using logistic regression with *competent* or *not competent* for maintaining preload, time to peak forces, and peak thrust force across 200 N, 400 N, and 800 N as the dependent variable, with *not competent* as the reference group. Again, demographic, anthropometric, and experience factors were independent variables. We first ran all models univariable, followed by multivariable models that included all independent variables as covariates while ensuring appropriate covariate selection based on causal relationships.

Models were stratified on whether independent variables were numerical or categorical. We presented beta-estimates with 95% confidence intervals (95%CI) for all confidence models and odds rations (OR) with 95%CI for all competent models. Results are reported using heat maps color-scaled by beta-estimates or OR, here we also present whether the finding was statistically significant, defined as *p* value < 0.05. Model assumptions were checked visually for normality of residuals, homogeneity of variance, linearity and influential observations. For multivariable models, we also checked collinearity using variance inflation factors, with collinearity defined as a factor > 5.0

All data analyses were conducted using R version 4.3 and R-Studio version 2023.12 [23], utilizing the *Tidyverse* programming language [24]. For information related to the study setup and completion (e.g., number of successful trials, use of contact type) please refer to the primary analysis [8].

## Results

Participants were nearly equally distributed across the years of study and sex. The median age of the participants was 24, with little variability (Table 1).

**Table 1:** Demographic, anthropometric and experience factors on 149 chiropractic students

Factors	N = 149
Sex [males, n (%)]	76 (51%)
Age [Years, Mean (SD)]	24 (3)
Height [cm, Mean (SD)]	173 (15)
Weight [kg, Mean (SD)]	74 (20)
Year of study [n (%)]	
1st year	47 (32%)
2nd year	47 (32%)
3rd year	55 (37%)
Time in FSTT® laboratory [hours, Mean (SD)]	58 (14)

**Fig. 2:**
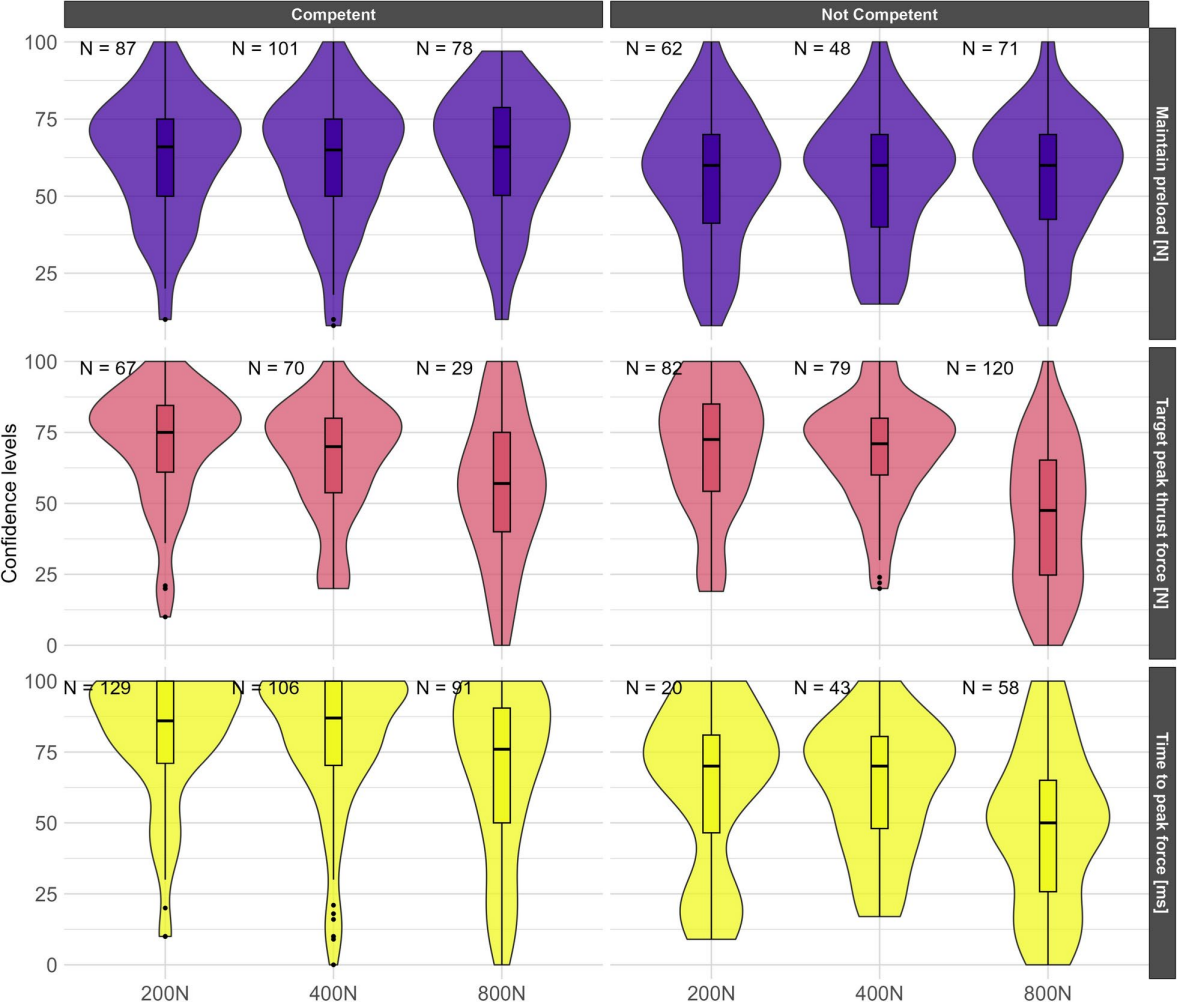
Students’ confidence in maintaining preload (± 75 N), achieving the target peak thrust force (± 75 N), and time to peak force under 150 ms stratified by being competent in achieving such characteristics

Participants generally had high confidence independent of whether or not they were able to maintain their preload and perform the lower peak thrust forces (200 N and 400 N). Notably, confidence levels became lower and more variable as the target peak forces increased to 800 N (Fig. 2).

**Fig. 3:**
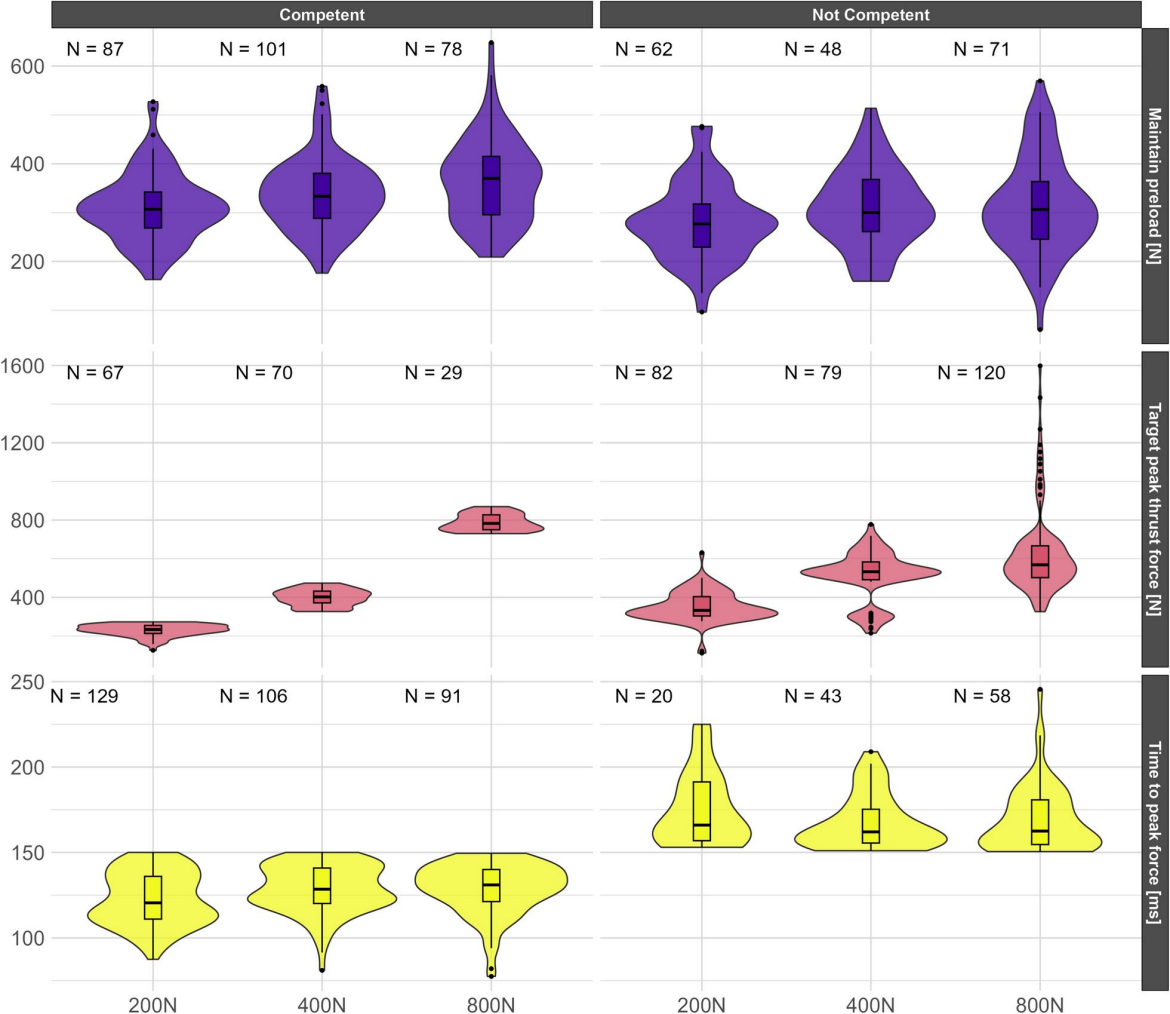
Students’ SMT force–time characteristics performed during their pre-established preload and across 200 N, 400 N, and 800 N peak thrust forces stratified by their ability to maintain preload (± 75 N), hit the peak force targets (± 75 N), and time to peak under 150 ms

Participants displayed substantial variability in all the SMT force–time characteristics. Generally, those who were competent showed less variability, particularly at lower peak force levels. Although very few participants were able to achieve the 800 N peak thrust force, they still performed the thrust in less than 150 ms; in fact, often much faster across all the target forces (Fig. 3).

### Associations with confidence

**Fig. 4:**
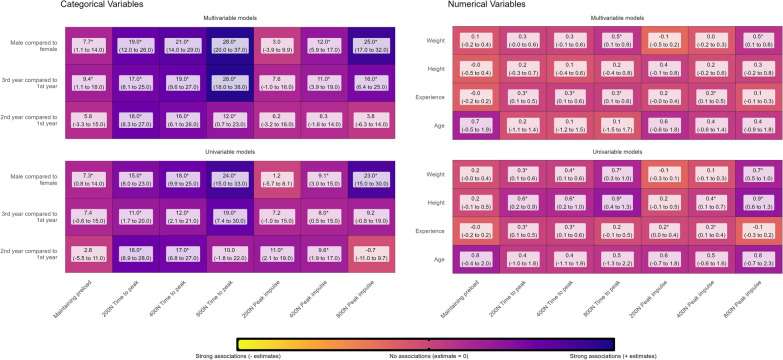
Associations between students’ demographic, anthropometric, and experience factors and level of confidence in performing SMT force–time characteristics of 200 N, 400 N, and 800 N peak thrust forces. Results from multivariable linear regression, categorical variables illustrate odds-ratios (95% confidence intervals), and numerical variables illustrate beta-estimates (95% confidence intervals), * = *p* value < 0.05

Based on both univariable and multivariable analyses, men were much more likely to be confident than females, especially in higher force ranges (i.e., 400 N and 800 N). Likewise, upper-year students (2nd and 3rd year) were generally more confident than 1st-year students (Fig. 4, categorical variables). In the univariable models, weight and height were generally associated with higher confidence. However, only weight at the 800 N force remained statistically significant in the multivariable models. Age was not strongly associated with confidence, whereas, time spent in the FSTT® lab, showed small increases in confidence levels (Fig. 4, numerical variables). All model assumptions were upheld.

### Associations with competence

**Fig. 5:**
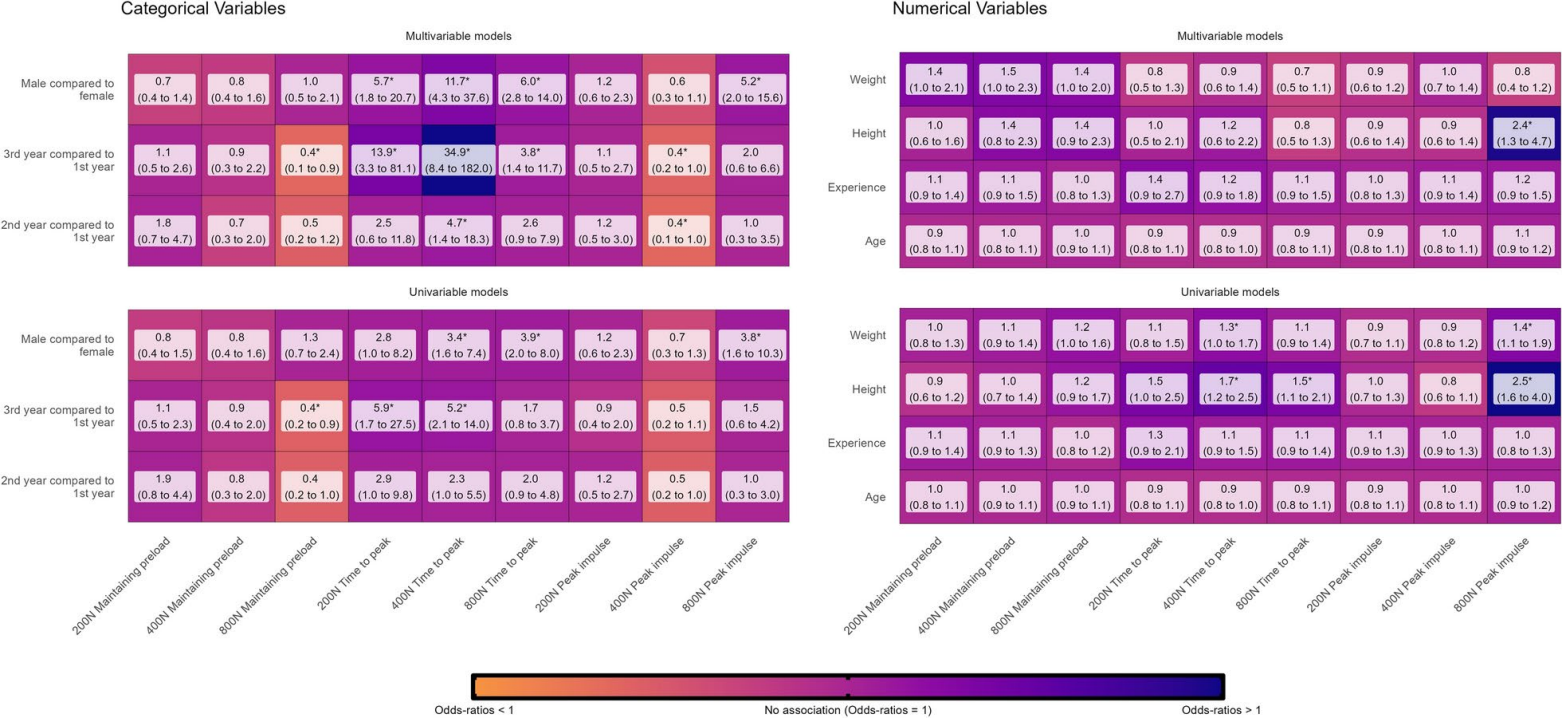
Associations between students’ demographic, anthropometric, and experience factors and their competence in achieving SMT force–time characteristics of 200 N, 400 N, and 800 N peak thrust forces. Results from multivariable logistic regression, categorical variables illustrate odds-ratios (95% confidence intervals), and numerical variables illustrate beta-estimates (95% confidence intervals),* = *p* value < 0.05

In the univariable models, males were more likely to achieve time-to-peak forces and provide 800N peak thrust forces. Likewise, in the multivariable models, the time to peak force showed consistently strong associations, and the likelihood of achieving 800N peak forces increased for males. There were no other strong associations between sex and other peak thrust forces or preload forces. Third-year students were substantially more likely to be faster than 1st-year students. The same pattern emerged with 2nd-year students compared to 1st-year students but with more uncertainty and less strong associations. Again, these associations were strongest in the multivariable models (Fig. 5, categorical variables).

Taller and heavier students were more likely to achieve time to 800 N peak and 800 N peak thrust forces. However, in the multivariable models, only height remained statistically significant for 800 N peak thrust force. No other associations were found to be strong with high certainty (Fig. 5, numerical variables).

## Discussion

Our study aimed to investigate the associations between demographic, anthropometric, and SMT experience factors and chiropractic students' confidence and competence in delivering specific SMT force–time characteristics. We found that confidence varied significantly, with males and students in later years in the chiropractic program showing higher confidence levels. Likewise, these groups also had higher odds of performing SMT with time to peak force below 150 ms, and achieve the 800 N peak thrust force. However, no other force–time characteristic showed a strong relationship with sex or year of study. Additionally, taller students, regardless of sex, had higher odds of achieving the 800 N peak thrust force, a target force that demonstrated the highest variability and posed the most challenge for students. Furthermore, while time spent in the FSTT® lab significantly influenced confidence, it did not translate into higher competence.

Improvement in chiropractic students’ confidence and competence in performing specific SMT force–time characteristics aligns with existing research, which indicates that students progressively develop confidence as they move into advanced stages of training and gain experience, particularly with more complex SMT techniques [19, 20]. Similar patterns are evident in medical education, where increased clinical exposure boosts confidence in skills such as bedside testing [25], and in physiotherapy, where confidence grows during clinical training [26]. While it is intuitive that more experience is related to more confidence, our previous analysis did not reveal strong associations between these two [8], and other factors (e.g., being male) were more important determinants of confidence than just time spent in the lab. Additionally, we found that, although, more SMT experience increased students’ confidence, it was not associated with improved competence. This suggests that competence may be shaped by factors beyond experience, and interventions might be needed to better align confidence with actual competence. The observation that males were more confident than females aligns with existing literature in medicine, where males often report higher confidence [27]. This trend is also reflected in chiropractic education, where studies have reported female students to be more likely to experience imposter phenomenon, highlighting gender-related disparities in perceived confidence [28].

Our study population had extensively used the FSTT® throughout their training (average of 58 ± 14 h during 7 months prior to data collection). While the evidence suggests that structured training and feedback mechanisms can reduce variability and improve force modulation, our findings indicate that the amount of experience did not significantly enhance competence in all SMT force–time characteristics. Time spent practicing on the FSTT^®^ appears to increase students' confidence, which is likely beneficial for clinical practice. However, more practice time does not seem to improve students' competence in modulating SMT force–time characteristics. It is also possible we observed a ceiling effect, with the current FSTT® training methods used, beyond which additional practice time does not improve performance of specific force targets. Although this could reflect a mismatch in teaching strategies and individual learning strategies, it remains unknown how SMT force–time characteristics affect clinical practice [29]. Further research is needed to assess whether using force-sensing devices in chiropractic curricula enhances clinical outcomes.

Additionally, despite the extensive use of FSTT^®^, students struggled to deliver higher target forces (i.e., 800N) and lacked confidence in doing so. While higher thrust forces are only introduced later in the chiropractic curriculum and the limited ability to achieve the 800 N thrust force observed could be related to limited practice, even students in the later years of their program struggled with achieving this target thrust force. This is concerning, as delivering such forces can be critical in certain clinical scenarios [30]. While being able to perform SMT with higher forces while maintaining a pre-established preload and with time to peak force below 150 ms may naturally develop during the initial years of clinical practice, the observed overconfidence among some students, particularly male and third-year students, highlights a potential risk. While speculative, this overconfidence could potentially have safety and effectiveness implications as some students may believe they are more competent in modulating SMT forces and provide too high or too low forces resulting in an adverse event or not enough for a clinical effect. This suggests that training programs must address both competence and realistic self-assessment. However, clinicians may simply provide “individualized” force–time characteristics that best fit their approach and technique independent of the target in question. Such findings are evident from a recent study in which three controlled case situations were emulated in a laboratory setting. Experienced chiropractors also showed substantial variability independent of the controlled setting, yet the chiropractors were mostly able to provide consistent force–time characteristic when re-tested [31].

From a clinical perspective, developing both competence and confidence to modulate SMT forces across a spectrum may be critical for optimizing patient outcomes. Our findings suggest that refining training to ensure that all students, regardless of their physical characteristics, feel adequately prepared to deliver SMT safely and effectively in clinical settings is essential. Given these results, chiropractic education might benefit from a more balanced approach that integrates both biomechanical and psychosocial components to produce well-rounded practitioners. For example, providing female and short students with additional strategies to achieve higher forces may support the development of their competence as well as their confidence.

Our findings highlight the need for further investigations into the factors influencing confidence and competence in chiropractic education. Future research should explore longitudinal designs to track how confidence and competence, and their relationship, evolve throughout training and into early clinical practice. Research should also examine whether targeted interventions, like resilience training or confidence calibration workshops, can help align students' confidence with their actual competence, as has been explored in other educational contexts [32].

### Methodological considerations

While this study offers valuable insights into the factors associated with chiropractic students' confidence and competence in modulating force–time characteristics when applying SMT to mannikins, several limitations must be acknowledged. First, the cross-sectional design only provides a snapshot of participants' confidence and competence at a single time point, limiting our ability to draw prognostic risk factor of demographic, anthropometric, and SMT experience factors on confidence and competence in delivering SMT with specific force–time characteristics. With this design, we are also limited in extrapolating our findings beyond the data collection testing scenario, specifically related to SMT competence.

Participant recruitment through convenience sampling may have introduced selection bias, as students with a greater interest in SMT or those who are more confident in their skills may have been more inclined to participate. This could have skewed the results toward higher confidence and competence levels, limiting the generalizability of the findings to all chiropractic students. Yet, if this is the case, the lack of a strong association between factors, such as experience, and competence is particularly concerning. The lack of competence observed may be related to the absence of real-time visual feedback, which students are typically accustomed to during SMT training, however, in real world practice, these parameters are not readily available.

Additionally, unmeasured confounding variables, such as psychological factors (e.g., stress, anxiety, or self-efficacy) or prior physical or mental fatigue as this study was conducted after their examinations, could have influenced both confidence and competence. These factors may impact students’ competence to perform SMT with the specific force–time characteristics and were not accounted for in the study. Lastly, as this study was conducted at a single institution, the findings may not be generalizable to chiropractic students in different cultural or educational environments, where training methods and curriculum focus may vary significantly.

## Conclusions

This study offers valuable insights into the factors influencing chiropractic students' confidence and competence in performing SMT with different specific force–time characteristics. We identified key associations with confidence and competence to reach specified target forces by incorporating demographic, anthropometric, and experience-related factors. Our findings highlight that males, students in later years of the chiropractic program, and students who spent more time using force-sensing devices had greater confidence in performing SMT with specific force–time characteristics. However, despite this increased confidence, only males and later-year students demonstrated higher competence in achieving faster time-to-peak forces and achieving 800N peak thrust force, and was not consistently associated with improved competence across other force–time characteristics. While SMT experience influenced confidence, it did not translate into higher competence levels.

## Data Availability

Data is available upon reasonable request, please contact data responsible at Canadian Memorial Chiropractic College Martha Funabashi (mfunabashi@cmcc.ca).

## Abbreviations

SMT: Spinal manipulative therapy
CMCC: Canadian Memorial Chiropractic Colleges
FSTT®: Force-sensing table technology
HAM®: Human analogue manikin
